# Design of Magnetic Gelatine/Silica Nanocomposites by Nanoemulsification: Encapsulation *versus in Situ* Growth of Iron Oxide Colloids

**DOI:** 10.3390/nano4030612

**Published:** 2014-07-31

**Authors:** Joachim Allouche, Corinne Chanéac, Roberta Brayner, Michel Boissière, Thibaud Coradin

**Affiliations:** 1 Institut Pluridisciplinaire de Recherche sur l'Environnement et les Matériaux (IPREM), Centre National de Recherche Scientifique (CNRS), Université de Pau et des Pays de l'Adour (UPPA), Unité Mixte de Recherche (UMR) 5254, Equipe de Chimie Physique (ECP), Technopôle Hélioparc Pau Pyrénées 2 avenue du Président Pierre Angot, PAU, 64053 Cedex 09, France; 2 Chimie de la Matière Condensée de Paris, UMR 7574, Université Pierre et Marie Curie, Bât F, 4 place Jussieu, and Collège de France, 11 place Marcelin Berthelot, Paris 75005, France; E-Mails: corinne.chaneac@upmc.fr (C.C.); thibaud.coradin@upmc.fr (T.C.); 3 Interfaces, Traitements, Organisation et Dynamique des Systèmes (ITODYS), Université Paris Diderot, UMR-CNRS 7086, Bâtiment Lavoisier, 15 rue Jean-Antoine de Baïf, Paris, 75205 Cedex 13, France; E-Mail: roberta.brayner@univ-paris-diderot.fr; 4 Equipe de Recherche sur les Relations Matrice Extracellulaire-Cellule (ERRMECe) EA 1391, Université de Cergy Pontoise–UFR Sciences et Techniques, 2 avenue Adolphe Chauvin BP222, Cergy Pontoise, 95302 Cedex, France; E-Mail: michel.boissiere@u-cergy.fr

**Keywords:** nanocomposites, gelatine, silica, iron oxide, nanoparticles, emulsion

## Abstract

The design of magnetic nanoparticles by incorporation of iron oxide colloids within gelatine/silica hybrid nanoparticles has been performed for the first time through a nanoemulsion route using the encapsulation of pre-formed magnetite nanocrystals and the *in situ* precipitation of ferrous/ferric ions. The first method leads to bi-continuous hybrid nanocomposites containing a limited amount of well-dispersed magnetite colloids. In contrast, the second approach allows the formation of gelatine-silica core-shell nanostructures incorporating larger amounts of agglomerated iron oxide colloids. Both magnetic nanocomposites exhibit similar superparamagnetic behaviors. Whereas nanocomposites obtained via an *in situ* approach show a strong tendency to aggregate in solution, the encapsulation route allows further surface modification of the magnetic nanocomposites, leading to quaternary gold/iron oxide/silica/gelatine nanoparticles. Hence, such a first-time rational combination of nano-emulsion, nanocrystallization and sol-gel chemistry allows the elaboration of multi-component functional nanomaterials. This constitutes a step forward in the design of more complex bio-nanoplatforms.

## 1. Introduction

The design of particles containing iron oxide colloids has become an intense field of research due to their large potentialities for biomedical applications [1,2,3]. The main challenges in this area include a precise control of the size dispersity of both magnetic colloids and encapsulating particles, a subtle balance between a high iron oxide loading and a good dispersion of entrapped colloids, as well as a suitable surface chemistry that should guarantee particle biocompatibility and, if necessary, allow further bio-functionalization [4,5,6].

Two alternative strategies have been explored to build-up such nanocomposites [7]. The first one relies on the preparation of the magnetic colloids, followed by surface coating with macromolecules [8,9,10,11,12] or sol-gel layers [13,14,15,16,17], interfacial polymerization via emulsion methods [18,19,20,21] or incorporation in a pre-formed host, including whole cells [22,23]. The second one involves the preparation of the host particles containing Fe^2+^ and/or Fe^3+^ ions, followed by the *in situ* precipitation of iron oxide [24,25,26,27].

In this context, we have previously proposed a new family of nanocomposites, named hybrid magnetic carriers (HYMAC), consisting of biopolymer/silica nanoparticles incorporating magnetic colloids [28]. As a first step, alginate/silica and gelatine/silica hybrid nanomaterials could be easily obtained by adapting traditional routes used in pharmaceutical science to design polymer nanoparticles [29,30]. These nanocomposites showed an enhanced thermal stability when compared to their biopolymer equivalents. Moreover, they could be up-taken by fibroblast cells and degraded intracellularly, without inducing rapid cell death. The first attempts to incorporate pre-formed magnetite colloids within alginate/silica nanocomposites via a spray-drying process were described, but the formation of lepidocrocite γ-FeOOH and fayalite Fe_2_SiO_4_ was observed, attributed to Fe^2+^ release during the aerosol thermal treatment [9]. Substitution of magnetite colloids by maghemite nanocrystals avoided fayalite formation, but some lepidocrocite was still present [31]. Lowering the process temperature led to a decrease in silica condensation, affecting the hybrid nanoparticle stability [32].

In this work, we have studied the possibility to prepare novel iron oxide/gelatine/silica nanoparticles following an emulsion route that takes place near room temperature [30]. This procedure is shown to be compatible with both encapsulation and *in situ* precipitation processes. The *in situ* approach allows the formation of iron oxide/gelatine nanoparticles that can be further coated with silica, leading to core-shell nanocapsules. In contrast, the stable encapsulation of pre-formed colloids requires the cross-linking of the gelatine network by silicates within the emulsion droplets, resulting in homogenous protein/silica nanocomposites. These two approaches therefore yield nanomaterials that differ in terms of iron oxide colloid size, loading and, hence, in magnetic properties. Moreover, they present different surface chemistry, as indicated by their stability in solution and suitability for further grafting of organic moieties. Overall, the key difference between these two approaches lies in the strength of the gelatine/iron oxide interactions that dictate the mode of addition of silica precursors. These results indicate that the design of multi-functional nanocomposites should take into account the intrinsic reactivity of each component, as well as possible interplays between them in order to find conditions that are compatible with their association. When these conditions are achieved, complex objects, such as gold/iron oxide/silica/gelatine systems, can be elaborated. This opens the route to the design of novel nano-platforms that would combine optical and magnetic properties together with biocompatibility and could therefore find applications as diagnostic and/or therapeutic nano-devices [33,34,35,36,37,38].

## 2. Nanocomposites Preparation Procedures

We have previously described the synthesis of hybrid gelatine/silica nanocomposites using a nano-emulsification approach [30]. With the aim of designing novel hybrid magnetic nanomaterials, we have tried to adapt this method to the incorporation of iron oxide colloids (see the Experimental Section for more details), either via an encapsulation route (Procedure 1, sample named NPGMSi-1) or via an *in situ* growth process (Procedure 2, samples named NPGM-2 and NPGMSi-2).

### 2.1. Procedure 1: Synthesis of NPGMSi-1 Nanoparticles

The first procedure is divided into three steps. The magnetite colloids are first prepared by a simple co-precipitation method. In order to obtain a stable ferrofluid at pH = 7, trisodium citrate is used to ensure steric stabilization of the magnetic colloids [39,40]. In the second step, the silica solution is directly incorporated in a gelatine/ferrofluid solution to initiate the silica condensation at pH = 7. The concentration of silica in the solution is adjusted to 20 mM in order to balance between effective gelatine/silica electrostatic interactions and the limited kinetics of silica polymerization [41]. Indeed, upon silicate addition, the viscosity of the solution tends to increase rapidly due to the rapid silica condensation favored by the pH conditions, the temperature (40 °C) and the presence of the gelatine amino groups [42,43]. A viscous, dispersed phase favors bigger droplet formation during emulsification. Therefore, the nanoemulsion has to be performed quickly after the introduction of the silica source in the gelatine/ferrofluid solution (third step).

After cooling the nano-emulsion, the biopolymer gelation occurs, and nanocomposite precipitation is produced by water extraction from the nanomaterial due to the introduction of acetone.

### 2.2. Procedure 2: Synthesis of NPGM-2 and NPGMSi-2 Nanoparticles

In the second procedure, gelatine nanoparticles are first prepared following the nano-emulsion method and re-suspended in an ethanolic solution of Fe^2+^ and Fe^3+^, so that iron cations can diffuse inside the polymer network. After adding the alkaline catalyst, crystallization of magnetic colloids occurs both within the gelatine nanoparticles and in the surrounding solution. Magnetic colloids precipitated in the solution were withdrawn by membrane filtration due to their low diameter. Finally, iron oxide/gelatine colloids could be mixed with a silicate solution, as previously described, leading to silica deposition on the particle surface.

The first procedure is expected to give rise to homogeneous hybrid silica/gelatine nanocomposites as silica formation occurs within the emulsion droplets. In fact, attempts to deposit a silica shell on gelatine particles encapsulating pre-formed magnetite colloids were unsuccessful due to rapid leaching of these magnetic nanoparticles out of the biopolymer network when put in contact with the silicate solution. On the contrary, leaching was not observed when Fe_3_O_4_ colloids were grown *in situ* in the gelatine nanoparticles, allowing the formation of core-shell structures.

## 3. Nanocomposite Characterization

### 3.1. Structural Characterization

**Figure 1:**
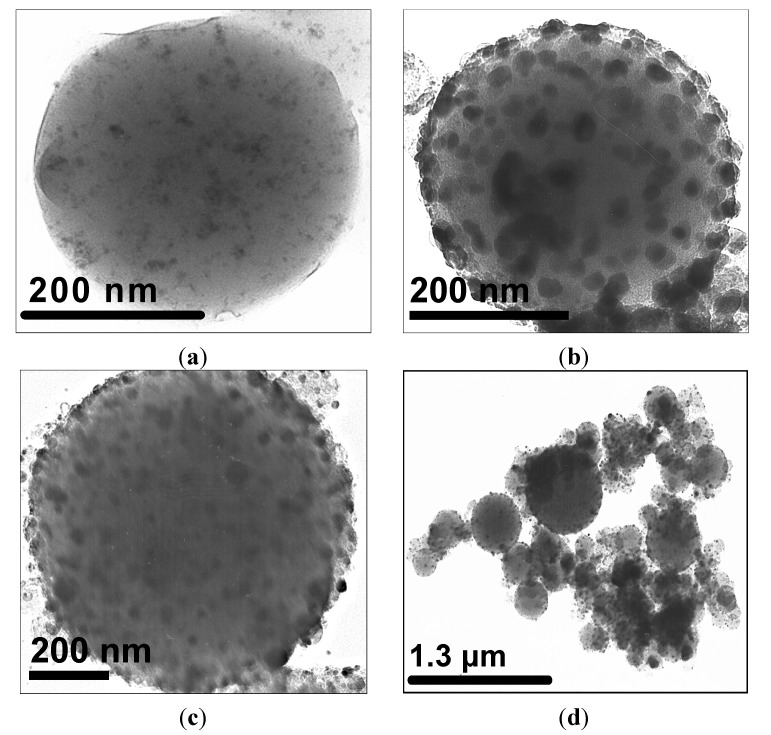
Transmission electron microscopy (TEM) images of: (**a**) NPGMSi-1; (**b**) NPGM-2; and (**c**,**d**) NPGMSi-2.

A transmission electron microscopy (TEM) image of an iron-oxide encapsulating silica/gelatine NPGMSi-1 nanoparticle is shown on Figure 1a. This particle presents a spherical shape with a smooth surface and well-dispersed dark colloids inside. As expected, the structure of the hybrid material is continuous, suggesting the presence of interpenetrating networks of both organic and inorganic compounds. As a comparison, Figure 1b,c shows TEM images of *in situ* modified NPGM-2 and NPGMSi-2 nanoparticles, respectively. The core-shell structure of NPGMSi-2 is not as clearly observable as for previously reported silica/gelatine nanoparticles [30]. The *in situ*-grown dark colloids are larger than those incorporated in NPGMSi-1 nanocomposites.

**Figure 2:**
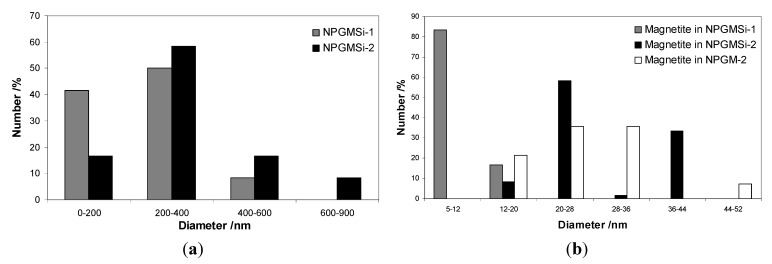
Size distribution of: (**a**) NPGMSi-1 and NPGMSi-2 nanocomposites; and (**b**) iron oxide colloids within NPGMSi-1, NPGM-2 and NPGMSi-2 nanocomposites from TEM analysis.

Dynamic light scattering measurements were carried out for the two types of hybrid materials, but estimations of the sizes were difficult, due to strong and large aggregates for NPGMSi-2 (Figure 1d) and the poor water stability of NPGMSi-1. The size distributions of NPGMSi-1 and NPGMSi-2 nanoparticles were evaluated by TEM statistical observations and presented in Figure 2a. The diameter distribution is slightly broader for NPGMSi-2 when compared to NPGMSi-1, but both exhibit an average diameter in the 200–400 nm range.

Energy dispersive spectrometry (EDS) analyses were performed to obtain the chemical composition of the two nanocomposites. First, the typical X-ray loss energies spectra (not shown) of NPGMSi-1 and NPGMSi-2 particles confirm the presence of silica for the two types of hybrid materials. However, the Fe, Si and C atomic contents for the two types of particles are very different. The atomic compositions are Fe:Si:C = 1.5:0.5:98 for NPGMSi-1 and 5.5:17:77.5 for NPGMSi-2. For NPGMSi-1, the initial atomic ratios were Fe:Si:C ≈ 5:5:90, but the preparation of particles requires a water-extraction step by acetone, which leads probably to an extraction from the initial particles of magnetite and silica compounds, as mentioned above. As a result, the Fe/C ratio decreases from 0.055 to 0.015 and the Si/C ratio from 0.055 to 0.005. For NPGMSi-2, the preparation conditions involve a composition of Fe:Si:C ≈ 30:10:60, which corresponds to much higher Fe/C (0.5) and Si/C (0.165) initial ratios. However, it was noticed that iron oxide precipitation occurs both within the particles and in the surrounding solution, explaining the lower final Fe/C (*i.e.*, iron oxide/gelatine) ratio of 0.071 found in the nanocomposites. This is confirmed by the EDS analysis of un-silicified NPGM-2 nanocomposites that indicate a ratio Fe/C ≈ 0.111. In parallel, the Si/C ratio ≈ 0.219 obtained by EDS suggests a higher silica content of the final material when compared to the initial mixture. However, this might reflect the surface sensitivity of this method, which overestimates the Si content for NPGMSi-2 nanocomposites exhibiting a gelatine-silica core-shell structure.

### 3.2. Magnetic Colloid Characterization

**Figure 3:**
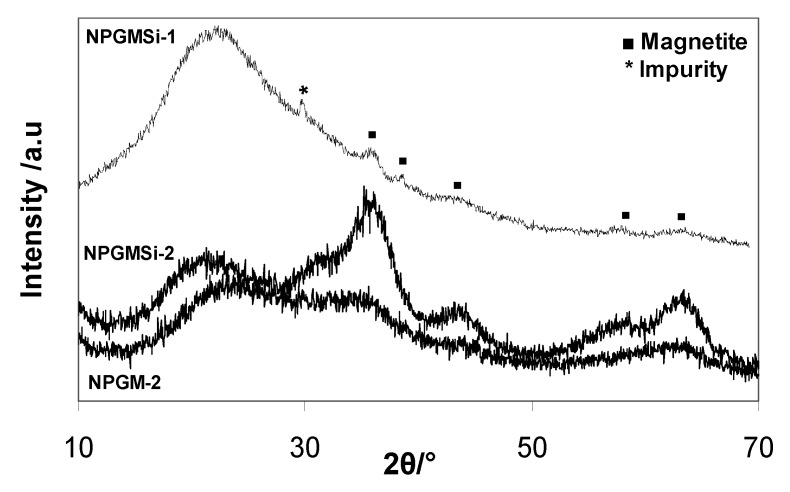
X-ray powder diffraction (XRD) diffractograms of NPGMSi-1, NPGM-2 and NPGMSi-2 nanocomposites.

The X-ray powder diffraction (XRD) data for both nanocomposites are shown in Figure 3. Due to the presence of a high percentage of amorphous materials (silica, gelatine) and low iron content, the diffraction patterns exhibit a low signal-to-noise ratio, but some characteristic diffraction peaks of the spinel iron oxide structure could be identified. For NPGMSi-1 and NPGMSi-2, diffraction peaks are broad, and their 2θ positions indicate the presence of magnetite Fe_3_O_4_ or maghemite γ-Fe_2_O_3_. Due to poor diffractogram resolution, only the mean crystallite size for the NPGMSi-2 sample could be inferred from X-ray line broadening yielding to a value of 3.5 nm. TEM image analysis was also used to estimate the size of incorporated colloids (Figure 2b). For NPGMSi-1, the sizes of magnetite colloids exhibit a maximum between 5 nm and 12 nm, corresponding to their initial (*i.e.*, as prepared) dimensions. For NPGMSi-2, the maximum is located between 20 nm and 28 nm. When compared to the mean diameter size obtained from XRD, we can conclude that all observed particles are, in fact, aggregates. Interestingly, the XRD data of the non-silicified NPGM-2 samples indicate that these nanocomposites mainly contain amorphous ferrihydrite (Figure 3). Hence, some crystallization of the iron oxide phase has occurred during the silicification step, probably due to the re-suspension of NPGM-2 particles in an aqueous solution at neutral pH [44].

**Figure 4:**
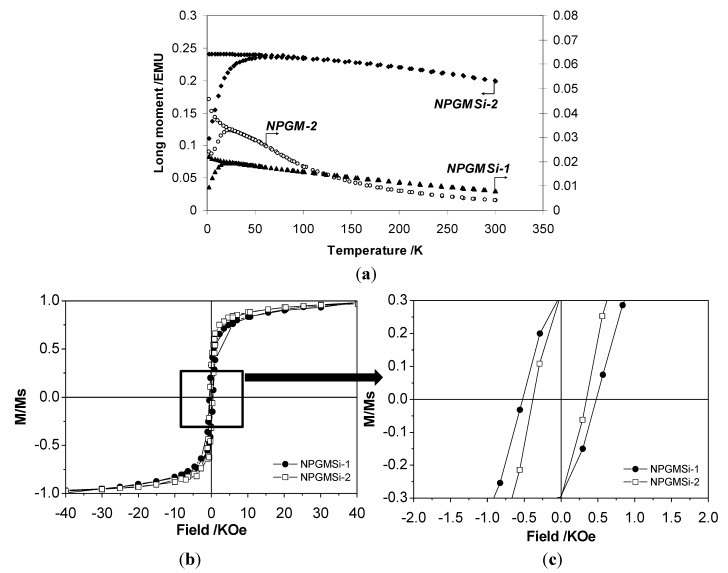
Magnetic properties of nanocomposites: (**a**) zero-field-cooling/field cooling (ZFC/FC)/ZFC susceptibility curves (*H* = 500 Oe); (**b**) low and (**c**) high magnification of normalized magnetization (*M*/*M*_S_) *vs.* magnetic field (*T* = 2 K).

In order to evaluate the magnetic properties of these hybrids particles, superconducting quantum interference device (SQUID) measurements were carried out. The features of zero-field-cooling/field cooling (ZFC/FC) susceptibility curves of these samples, reported in Figure 4a, indicate a superparamagnetic behavior for both nanocomposites. NPGMSi-1 and NPGMSi-2 exhibit blocking temperature values of *T*_B_ ≈ 26 K and *T*_B_ ≈ 55 K, respectively.

Such a difference in *T*_B_ value may be due to the aggregation of magnetic colloids and inter-particle magnetostatic interactions [45,46,47], as suggested by TEM and XRD analyses. In parallel, NPGM-2 nanoparticles showed a blocking temperature *T*_B_ ≈ 27 K, lower than that of NPGMSi-2, in agreement with the lower crystallinity of the iron oxide phase [45].

The superparamagnetic behavior of the nanocomposites could be confirmed by hysteresis loop measurements at room temperature and below the blocking temperature. All samples are paramagnetic at room temperature (curves not shown), but NPGMSi-1 and NPGMSi-2 exhibit a ferromagnetic behavior (Figure 4b) at 2 K involving a hysteresis loop, as shown in the highly magnified Figure 4c. This is a typical feature of small-sized ferromagnetic nanoparticles, where superparamagnetism induces a ferromagnetic-paramagnetic transition occurring below the Curie temperature. Corresponding coercivities (*H*_c_) and squareness (*S*) values for the two samples are *H*_c_ = 500 Oe and *S* = 0.23 for NPGMSi-1, and *H*_c_ = 400 Oe and *S* = 0.22 for NPGMSi-2. The slightly lower *H*_c_ value for NPGMSi-2 may arise from the presence of silica, as these results are very close to γ-Fe_2_O_3_ nanoparticles (5–9 nm) trapped in a silica xerogel [48].

### 3.3. Nanoparticle Surface Modification

One of the great advantages of silica materials is their easy and versatile chemical modification by organic moieties. Indeed, silicon alkoxides are widely used to functionalize silica surfaces as films, particles or pores. Foreseeing further developments of hybrid magnetic biopolymer/silica nanoparticles for *in vivo* applications, where the stability in physiological fluids and/or organ targeting will require surface functionalization [49], we have evaluated the suitability of NPGMSi nanocomposites for surface modification. We selected mercaptopropyltriethoxysilane (MPTS) as a model silicon alkoxide, since the thiol group allows further bio-functionalization, while it does not interfere significantly with the hydrolysis/condensation reactions. With this aim, the hybrid particles were put in contact with MPTS in an anhydrous organic solvent (toluene) to favor the interaction between the silanols located at the surface of the particles and MPTS, while avoiding alkoxide polymerization.

To easily visualize the efficiency of the grafting process, modified nanocomposites were put in contact with a suspension of a pre-formed gold colloid suspension in ethanol.

**Figure 5:**
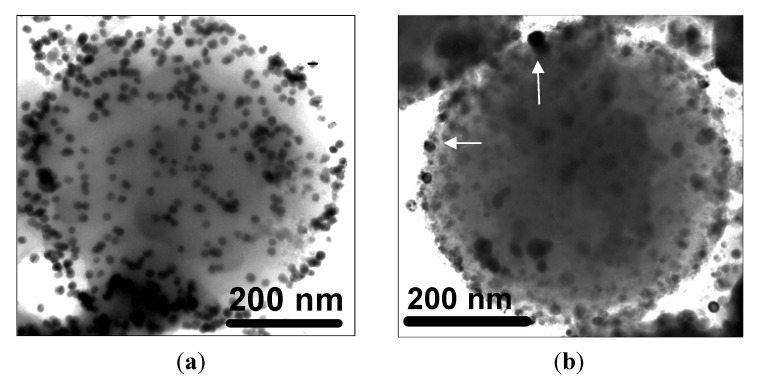
TEM images of: (**a**) Au-coated NPGMSi-1; and (**b**) Au-coated NPGMSi-2 (white arrows indicate Au colloids).

NPGMSi-1 particles could be dispersed in toluene, and the grafting reaction could be performed over 2 h without precipitation. After washing, the modified particles were reacted with gold nanoparticles. Corresponding TEM images (Figure 5a) show that the initial nanocomposites have been preserved and are now decorated with high contrasting dark gold colloids (diameter *ca*. 20 nm) deposited on their surface. EDS measurements (not shown) confirm the presence of Au and S atoms. In contrast, attempts to perform the same reaction with NPGMSi-2 were hindered by the formation of large aggregates when the nanocomposites were mixed with toluene. As a consequence, only a few gold nanoparticles could be observed on the MPGMSi-2 surface by TEM (Figure 5b).

## 4. Discussion

Main features of the two kinds of magnetic nanocomposites that were elaborated and studied in this work are gathered in Table 1. NPGMSi-1, obtained via the encapsulation of pre-formed iron oxide colloids, exhibits a bi-continuous structure with low silica and magnetite/maghemite content. The magnetic colloids are of a well-defined size and composition and lead to the superparamagnetic behavior of the nanocomposites above *ca*. 30 K. NPGMSi-1 is stable in organic solvents, allowing efficient surface modification. NPGMSi-2, obtained via the *in situ* crystallization of iron oxide, consists of a gelatine/silica core-shell structure with significant silica and magnetite/maghemite content. The iron oxide colloids tend to agglomerate, resulting in an increase of the blocking temperature due to magnetostatic inter-particles interactions [46]. NPGMSi-2 easily aggregates, both in water and in organic solvents, limiting surface accessibility to grafting agents.

**Table 1:** The main characteristics of NPGMSi magnetic nanocomposites, including NPGM diameter (*d*_NPGM_), structure and surface accessibility, Fe, Si and C concentrations, iron oxide colloid diameter (*d*_Fe_3_O_4__) and blocking temperature (*T*_B_).

Sample name	*d*_NPGMSi_^a^/nm	Structure	Surface accessibility	Fe ^b^/% At	C ^b^/% At	Si ^b^/% At	*d*_Fe_3_O_4__^c^/nm	*T*_B_^d^/K
NPGMSi-1	250 (150)	Bi-continuous	Good	1.5	98.0	0.5	9 (3)	26
NPGMSi-2	350 (200)	Core-shell	Poor	5.5	77.5	17.0	28 (8)	55

^a^ From TEM (standard deviation); ^b^ from energy dispersive spectrometry (EDS); ^c^ from TEM (standard deviation); and ^d^ ± 5 K (from superconducting quantum interference device (SQUID) measurements).

These differences allow the identification of the advantages and limitations of the two approaches. The encapsulation route benefits from the control of the magnetic colloids synthesis, and, therefore, leads to a well-defined particle size. However, the interactions between the host network and the encapsulated colloids are weak, so that these can be easily released when the nanocomposites are re-dispersed in solution. It is worth noting that the presence of citrate stabilizers on the magnetite surface may contribute to this poor affinity of the gelatine network for the magnetic colloids. On the contrary, the *in situ* crystallization approach is more difficult to control, but results in a stronger bonding of the iron oxide colloids to the biopolymer network. In fact, several studies indicate that iron ions have a good affinity for carboxylate groups present in poly-saccharide and protein chains, leading to an efficient anchorage of the grown iron oxide nanocrystals within the hydrogel. This difference in magnetic colloid retention also dictates the mode of association of the gelatine nanoparticles with silica. For NPGMSi-1, silicate introduction should be made within the emulsion droplets, and this limits the inorganic precursor concentration, because of the strong silicate/gelatine interactions. In contrast, NPGM-2 iron oxide/gelatine particles are stable enough to allow the deposition of a silica coating. However, in contrast to previous reports on silica/gelatine nanocomposites, the resulting NPGMSi-2 has a strong tendency to aggregate. Together with the observation that the silica coating is not easily identified on corresponding TEM images, it can be proposed that the presence of iron oxide colloids on the gelatine particle surface may interfere with the silicate condensation process.

On the basis of these results, it appears that the *in situ* growth process is the most promising route to design gelatine-based HYMAC. Optimization of the procedure will imply a better control of the Fe^2+^/Fe^3+^ stoichiometry within the polymer network to obtain a better control of colloid sizes. As the thickness of the silica shell was shown to increase with the silicate concentration [30], it should be possible to fully coat the magnetic colloids located on the gelatine surface and, hence, to obtain stable nanocomposites. In parallel, the encapsulation route may be suitable for other biopolymers that would interact more significantly with iron oxide colloids, to limit leaching processes, and less strongly with silicates, allowing the increase of silicate concentration in the emulsion droplets.

Finally, it is worth noting that, in contrast to previous data on alginate/silica nanocomposites [28,31], no evidence was found for the presence of fayalite or lepidocrocite phases within gelatine-based systems, in agreement with the mild temperature conditions of the nanoemulsion process.

## 5. Experimental Section

### 5.1. Nanocomposite Preparation by Encapsulation

**Scheme 1:**
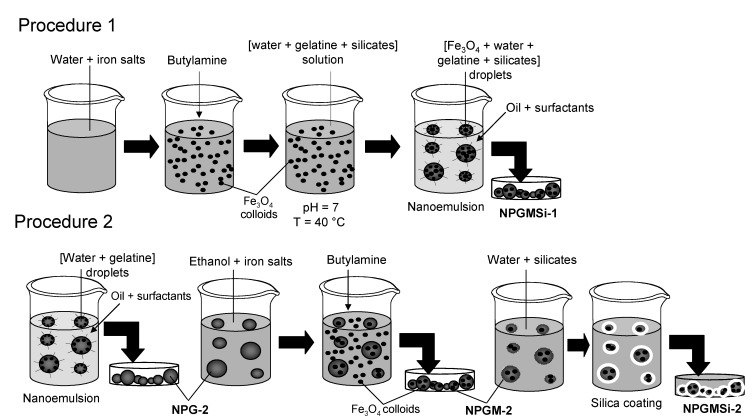
Illustration of the experimental procedures for the formation of iron oxide/silica/gelatine nanocomposites: Procedure 1 (encapsulation) and Procedure 2 (*in situ* growth).

Magnetite Fe_3_O_4_ colloids (Procedure 1 in Scheme 1) were prepared as follows [50]: A 10-mL chloride solution containing 0.5 M of FeCl_2_ and FeCl_3_ (both from Sigma Aldrich, Saint-Quentin Fallavier, France) in demineralized water ([Fe^2+^]:[Fe^3+^] = 2:1) was prepared, and 2 mL of butylamine was quickly poured in the solution under magnetic stirring, yielding colloid formation. 0.5 g of trisodium citrate (Sigma Aldrich) were then added, and the pH was adjusted to 7 with concentrated HCl to obtain a stable dispersion [39,40]. From TEM observations, the resulting colloids were 5–10 nm in diameter.

Nanocomposite NPGMSi-1 was prepared by nanoemulsification. The method used was based on our previous publication, but with some modifications [30]. First, 2 g of gelatine (Type A, 300 Bloom, Sigma Aldrich, isoelectric point (*p*I) = 8) were dissolved in a mixture of 5 mL of demineralized water and 5 mL of magnetite suspension at 40 °C. Concurrently, an oily phase composed of 0.12 g of Span 80 and 0.12 g of Tween 80 (both from Sigma Aldrich) dissolved in 5 mL of isooctane (Fluka^®^ from Sigma Alfrich Group) was equilibrated at 40 °C.

Then, 1 mL of a 200 mM solution of sodium silicate (6.25 M, 27% SiO_2_, 14% NaOH, Riedel de Haën^®^ from Sigma Alfrich Group) previously acidified to pH = 7 with HCl 3 M was poured in the gelatine solution at 40 °C in order to initiate the silica condensation. After 1 min of magnetic stirring, 2 mL of the as-prepared solution was poured in the oily phase and sonicated during 5 min using an ultrasonic probe (Measuring and Scientific Equipment (MSE) London, UK) to form a water-in-oil nanoemulsion. The emulsion was quickly cooled to 5 °C in an ice/water bath before adding 50 mL of acetone pre-equilibrated at 5 °C in order to precipitate the NPGMSi-1 nanocomposites. Nanoparticles were then filtered, washed five times with acetone in order to remove the surfactant and organic phase and left to dry in air.

### 5.2. Nanocomposite Preparation by In Situ Growth (Procedure 2 in Scheme 1)

First, gelatine nanoparticles were prepared using the above-described nanoemulsification method, except for the absence of the Fe_3_O_4_ colloids. These nanoparticles were then redispersed by sonication in a solution of FeCl_2_ and FeCl_3_ in ethanol ([Fe^2+^] + [Fe^3+^] = 0.5 M, to [Fe^2+^]:[Fe^3+^] = 2:1). After 30 min of magnetic stirring, 2 mL of butylamine were added to precipitate the magnetite colloids. Such a procedure allows *in situ* crystallization of iron oxide both in solution and within the gelatine particles (NPGM-2). After 10 min of stirring, the suspension was filtered on a 0.2-μm filter (Whaterman^®^ from GE Healthcare Life Sciences, Velizy-Villacoublay, France) to recover only the iron oxide/gelatine nanocomposites and washed five times with acetone.

NPGM-2 particles were re-dispersed in deionized water at 5 °C using an ice/water batch and added to a 20-mM solution of sodium silicate at pH = 7. The mixture was left to react for 1 h under mild magnetic stirring to form the silica/iron oxide/gelatine composite particles (NPGMSi-2). The particles were then filtered, washed five times with deionized water and washed another five times with acetone before drying in air.

### 5.3. Gold-Modified Nanocomposites

In order to evaluate the suitability of the magnetic nanocomposites for future imaging/targeting applications, NPGMSi-1 and NPGMSi-2 nanocomposites were modified by grafting thiol groups on the nanoparticles surface. 200 mg of nanoparticles were dispersed in anhydrous toluene, and five drops of MPTS (Sigma Aldrich) were poured in the suspension. The reaction was left to occur 2 h under magnetic stirring before filtering, washing five times with toluene and drying in air.

The modified nanocomposites were dispersed in 10 mL of ethanol, and 3 mL of Au metallic nanoparticles prepared by the citrate method [51] (*ca*. 20 nm in diameter from TEM) were added and left to react for 1 h. The resulting gold/iron oxide/silica/gelatine hybrid nanocomposites were recovered by filtration, washed five times with ethanol and dried in air.

### 5.4. Characterization Techniques

TEM of particles directly deposited on a carbon-coated copper grid was performed on a JEOL 100 CX microscope (Tokyo, Japan) working at 120 kV.

X-ray EDS was performed using an energy dispersive X-ray analysis (EDAX) system equipped with a super ultra-thin window (SUTW) connected to a JEOL JSM 6100 scanning electron microscope (Tokyo, Japan). The powder was placed on a copper-coated aluminum stub. Measurements were performed at 3 kV, and atomic compositions were obtained with Genesis software (EDAX Inc., Mahwah, NJ, USA).

XRD patterns were recorded using Cu Kα radiation. The diffractometer was calibrated using a standard Si sample. The counting time was 30 s per step of 0.05° 2θ. The mean crystallite size was estimated using the Scherrer equation.

Magnetic measurements were performed using a Quantum Design MPMS-5S SQUID magnetometer (San Diego, CA, USA) in the 2–300 K temperature range.

## 6. Conclusions

This work represents a step forward in the design of multi-functional hybrid magnetic nano-carriers. Whereas the formation of biopolymer/silica was previously easily achieved from alginate and gelatine, the incorporation of iron oxide magnetic colloids is revealed to be more challenging. If examples of (bio)-polymer or silica particles containing superparamagnetic nanoparticles are numerous [7,8,9,10,11,12,13,14,15,16,17,18,19,20,21,22,23,24,25,26,27], the elaboration of magnetic bio-hybrid nanocomposites has only been reported once, with limited success [28]. Here-described iron oxide/silica/gelatine nanomaterials are much closer to targeted devices, although improvements are still necessary in terms of chemical and physical stability. Moreover, surface modification experiments demonstrate that further grafting of the particles is possible, opening the route to the bio-functionalization of these nanomaterials.

Indeed, the design of multi-functional materials relies on the possibility of ensuring the compatibility of several components that may exhibit different reactivity and stability. However, the domain of reaction conditions that is adapted to the association of multiple components tends to narrow with increasing system complexity, requiring on exploring several alternative synthetic pathways. In our particular system, we had to deal with a system consisting of biological macromolecules, inorganic/organometallic molecular precursors and metal oxide colloids, each of which interact significantly with the two others. Moreover, these components had to be assembled at the nanoscale. Thus, the success of our approach has involved a combination of nanoemulsion, nanocrystallization and sol-gel techniques. Such versatility allows the design of quaternary gold/iron oxide/silica/gelatine nanocomposites. In our opinion, the here-demonstrated feasibility of such complex objects should constitute a useful basis for the future developments of bio-nanoplatforms [52,53,54].
